# Migraine-specific quality of life questionnaire and relapse of medication overuse headache

**DOI:** 10.1186/s12883-015-0339-8

**Published:** 2015-05-21

**Authors:** Stefano Caproni, Elisa Bianchi, Letizia M. Cupini, Ilenia Corbelli, Ettore Beghi, Paolo Calabresi, Paola Sarchielli, SAMOHA Study Group

**Affiliations:** Clinica Neurologica, Azienda Ospedaliera - Università di Perugia, Perugia, Italy; IRCCS Istituto di Ricerche Farmacologiche “Mario Negri”, Milan, Italy; Centro Cefalee e Malattie Cerebrovascolari, Ospedale S. Eugenio, Rome, Italy; IRCCS Fondazione “S. Lucia”, Rome, Italy

**Keywords:** MOH, MSQ, Relapse

## Abstract

**Background:**

The management of Medication overuse headache (MOH) represents a difficult challenge for clinicians and headache experts, particularly for the responder rate after a successful withdrawal treatment. The purpose of this study was to investigate the role of demographic and clinical characteristics as well as the score of Migraine-Specific Quality of Life Questionnaire (MSQ), Migraine Disability Questionnaire and Leeds Dependence Questionnaire in predicting a response after a successful withdrawal treatment in patients with MOH.

**Methods:**

This ancillary study is part of a randomized trial that demonstrated the safety and the efficacy of a 3-month treatment with sodium valproate (VPA) (800 mg/day vs placebo) in MOH. Demographic and clinical characteristics and questionnaire results were obtained from the entire sample.

**Results:**

A significant correlation was found only between MOH relapse and the total MSQ score, the Role Preventive sub-scale and the Emotional Function sub-scale, suggesting a poorer quality of life in non responders.

**Conclusion:**

A high MSQ score could be associated with a poor short-term outcome in MOH patients after a successful treatment with detoxification followed by a new treatment.

## Background

Medication-overuse headache (MOH) is a chronic headache having a relevant impact in clinical practice, with a prevalence of 1-2 % in the general population [1, 2]. The primary headache leading to MOH is migraine in most cases. The management of MOH represents a difficult challenge for clinicians and headache experts [3]. Indeed, even in case of a successful withdrawal treatment, the relapse rate of MOH ranges between 24 % and 43 % (40 % during the first year after withdrawal) [4]. Most studies indicate that the relapse usually occurs within few months after detoxification [5].

In recent years an increasing number of studies focused on the predictors of relapse of MOH, but no reproducible results have been achieved. While in a 4-year follow-up no significant predictors were found [6], in three studies with 1-year follow-up, predictors of relapse were, respectively, a long duration of migraine before medication overuse, a higher frequency of migraine after withdrawal, and a great number of previous preventive treatments [7], male sex, intake of combination analgesics after withdrawal, nicotine and alcohol consumption [8], the type of primary headache and the type of the overused medication [9]. Generally, even if proper instructions and appropriate surveillance are considered necessary to avoid relapse [4], no definite clinical predictors for the management of MOH patients are available.

The complexity ofMOH often depends on the comorbidities that are reported more frequently compared to what happens for episodic headaches. Thus, MOH patients show increased disability, reduced mood and social impairment compared with episodic migraineurs, leading to a worsening in their quality of life [3]. Recently, a high score measured by a self-reported quality of life tool resulted a predictor for a poor outcome of MOH patients [10]. In the last years patient-reported outcome measures have been applied to MOH to evaluate the consequence of this disorder on patients’ daily lives. Therefore, as with migraine, self-reported quality of life questionnaire’s scores could be considered as secondary endpoints in clinical trials along with clinical characteristics of MOH. Moreover, they could be investigated as outcome and relapse predictors.

Recently, the Sodium vAlproate in the treatment of Medication Overuse HeadAche (SAMOHA) study demonstrated the efficacy and safety of sodium valproate (VPA), after detoxification, in the short-term treatment of MOH patients with a history of migraine without aura. Moreover, patients treated with VPA reported a significantly higher improvement in the MSQ at the end of the treatment, compared to controls, whereas this difference was non-significant at the 3-month follow-up visit [11]. In light of this, we analysed data obtained from patients enrolled in the SAMOHA study, in order to investigate the role of the basal demographic and clinical characteristics along with disability, dependence and quality of life as outcome and response predictors.

## Methods

### Standard protocol approvals, registrations, and patient consents

As an ancillary study, the protocol was submitted for approval to the Ethics Committees of each participating center. The SAMOHA trial was registered on the European Union Drug Regulating Authorities Clinical Trials website (EudraCT code 2007-006773-92; https://www.clinicaltrialsregister.eu/ctr-search/trial/2007-006773-92/IT). The SAMOHA study was conducted in accordance with the Declaration of Helsinki and its amendments (Seoul, October 2008). All patients provided their written consent to participate in the study.

### Subjects

The SAMOHA study was a multicenter, randomized, double-blind, placebo-controlled study that randomized 88 MOH patients for a 3-month treatment period with VPA (800 mg/day) or placebo after a 6-day outpatient detoxification regimen, followed by a 3-month follow-up [12].

### Statistical analysis

This report describes post-hoc secondary data analysis from the SAMOHA study. Analyses were performed in the intent-to-treat population. Missing data were handled using the last observation carried forward approach (LOCF) in patients with at least one assessment. Demographic (sex, age), clinical (body mass index, comorbidity, surgery) and headache characteristics (frequency, intensity, total and MOH duration, overused drugs), the Migraine Specific Quality of Life Questionnaire (MSQ) [12] score, and the scores of the three sub-scales– Role Restriction (RR), Role Preventive (RP) and Emotional Function (EF), − as well as the Migraine DisAbility queStionnaire (MIDAS) [13] and the Leeds Dependence Questionnaire (LDQ) [14] scores, were compared at baseline in Responders (patients achieving ≥50 % reduction in the number of days with headache per month) and Non-Responders at the 24-week visit (R and NR, respectively). Comparisons between R and NR are reported as count and percentage, or median and interquartile range (IQR). Differences between the two groups were tested using the Fisher’s exact test or the Wilcoxon-Mann–Whitney test. Data resulting significant in univariate analysis were also adjusted for treatment using logistic regression models. Results are reported as adjusted odds ratios (OR) with 95 % confidence intervals (CI). All tests were two-tailed, and the significance level was set at 5 %. All analyses were performed using the SAS statistical analysis system version 9.2 (SAS Institute Inc., Cary, NC, USA).

## Results

The LOCF imputation method allowed the identification of 31 R and 51 NR at 24 weeks (3 months after the discontinuation of treatment). The 3-month proportion of patients achieving ≥50 % reduction in the number of days with headache per month in the entire sample was 34.1 %, while this rate was 37.8 % at the 24-week visit. A significant difference between R and NR was found for the total MSQ score (median 33.0, IQR 24.0-41.0, vs. 40.0, IQR 27.0-50.0, *p* = 0.0180), the RP sub-scale (median 6.0, IQR 5.0-9.0, vs. 9.0, IQR 6.0-13.0, *p* = 0.0195) and the EF sub-scale (median 5.0, IQR 2.0-7.0, vs. 8.0, IQR 4.0-12.0), while the RR sub-score and the total MIDAS and LDQ total scores as well as the other demographic, clinical and headache variables were evenly distributed in the two groups (Table 1). After adjusting for treatment, the association between MOH relapse and the total MSQ, the RP sub-scale and the EF sub-scale, was still statistically significant. The adjusted ORs were: 1.04 (95 % CI 1.01 – 1.08, p = 0.0249) for the total MSQ, 1.15 (95 % CI 1.03 – 1.29, p = 0.0163) for the RP sub-scale, and 1.14 (95 % CI 1.02 – 1.28, p = 0.0257) for the EF sub-scale.

**Table 1:** Comparison of baseline demographic, clinical and headache characteristics, total MSQ, EF, RP and RR, MIDAS and LDQ, between responders and non-responders at the 24-week visit

	Non-responders (51)	Responders (31)	
	*n* (%)	*n* (%)	*p*-value
Sex			
F	41 (80.4)	24 (77.4)	0.7475
M	10 (19.6)	7 (22.58)	
Age class (years)			
18-34 years	8 (15.7)	5 (16.1)	0.6547
35 - 44 years	20 (39.2)	10 (32.3)	
45 - 54 years	18 (35.3)	10 (32.3)	
55 - 64 years	5 (9.8)	6 (19.3)	
BMI class			
(<18) Underweight	2 (4)	1 (3.2)	0.4466
(18–24.9) Normalweight	32 (64)	17 (54.8)	
(25–29.9) Overweight	10 (20)	11 (35.5)	
(>30) Obese	6 (12)	2 (6.5)	
Missing	1		
Comorbidity			
No	36 (70.6)	25 (80.7)	0.3117
Yes	15 (29.4)	6 (19.3)	
Surgery			
No	15 (30.6)	7 (23.3)	0.4836
Yes	34 (69.4)	23 (76.7)	
Missing	2	1	
Headache days/month			
15-25	22 (43.1)	17 (54.8)	0.3036
>25	29 (56.9)	14 (45.2)	
Headache intensity			
Slight	0 (0)	3 (9.7)	0.0639
Moderate	20 (39.2)	9 (29)	
Severe	31 (60.8)	19 (61.3)	
Headache duration (years)			
0 - 10 years	8 (15.7)	3 (9.7)	0.8787
11 - 20 years	12 (23.5)	7 (22.6)	
21 - 30 years	16 (31.4)	11 (35.5)	
>30 years	15 (29.4)	10 (32.2)	
MOH duration (years)			
<1 year	7 (13.7)	3 (9.7)	0.6263
1-3 years	13 (25.5)	11 (35.5)	
3-5 years	13 (25.5)	5 (16.1)	
>5 years	18 (35.3)	12 (38.7)	
Over used drugs			
Analgesics > 14 days	20 (39.2)	9 (29)	0.3617
Analgesicscombinations > 9 days	5 (9.8)	7 (22.6)	
Drugcombinations > 9 days	15 (29.4)	7 (22.6)	
Triptancombinations > 9 days	11 (21.6)	8 (25.8)	
	Median (IQR)	Median (IQR)	*p*-value
MSQ	40.0 (27.0 - 50.0)	33.0 (24.0 - 41.0)	0.0180
EF	8.0 (4.0 - 12.0)	5.0 (2.0 - 7.0)	0.0330
RP	9.0 (6.0 - 13.0)	6.0 (5.0 - 9.0)	0.0195
RR	23.0 (16.0 - 27.0)	18.0 (15.0 - 24.0)	0.1188
MIDAS	70 (24.0 - 96.0)	38.0 (15.0 - 75.0)	0.1119
LDQ	7.0 (5.0 - 14.0)	8.0 (7.0 - 13.0)	0.5031

BMI: Body Mass Index

MSQ: Migraine-Specific Quality of Life Questionnaire

EF: Emotional Function

RP: Role Preventive

RR: Role Restriction

MIDAS: MIgraine DisAbility queStionnaire

LDQ: Leeds Dependence Questionnaire

IQR: Interquartile range

## Discussion

Our findings suggest that only a high MSQ score is associated with a poor short-term outcome after a successful treatment with detoxification followed by a new treatment. In particular, the most significant difference was found for the RP and EF sub-scales, that measure the degree to which performance of normal activities is prevented or completely interrupted by headache and, respectively, the impact of symptoms on emotional well-being. These aspects can be considered the very disabling aspects of headache compared to those measured by the RR sub-scale. The MSQ score can be considered an indicator of headache severity self-reported by the patients, assessing the most disabling impact of MOH in daily life. Thus, MOH patients reporting a high MSQ score before treatment should be considered at high risk of short-term treatment failure. This result can be of substantial help for clinicians in the management of this type of headache. Of note, the lack of correlation between response and the MIDAS and the LDQ scores indicates that quality of life rather than functional disability and dependence is likely to have an influence on the response to any new treatment in patients with MOH.

All the other variables were evenly distributed in the two groups. These findings are in contrast with previous studies [6–9], perhaps because of the high variability among study populations. Further main reasons for non-reproducible results are probably differences in protocol, treatment and follow-up.

On this background, our results can represent a useful starting point for the general management of MOH. In fact, it is possible to argue that if a high MSQ score is reported, even in absence of relevant comorbidities, the patient should be considered at high risk of short-term treatment failure. Moreover, MSQ is easy to obtain, patient-friendly, reproducible, and suitable for short- and long-term follow-up.

Our study has some limitations. First, the small sample size prevents definitive results on the demographic and clinical characteristics we investigated. Second, our study analyzed a short-term follow-up, thus the correlation between MSQ score and response to treatment in the long-term was not assessed. Third, as we did not adjust for multiple comparisons, the possibility that our results are chance findings cannot be excluded. A prospective study is thus needed to compare MSQ with the clinical characteristics of patients suffering from MOH (including the treatment protocols) to predict short- and long-term specific outcomes.

## Conclusions

In conclusion, our findings suggest that a high MSQ score may be associated to a negative outcome of MOH patients after detoxification followed by a new treatment. Larger studies with longer follow-up are needed to clarify this issue given that their results could lead to a better management of MOH patients.

## Abbreviations

EF: Emotional function
IQR: Interquartile range
NR: Non-responders
MOH: Medication-overuse headache
MSQ: Migraine-specific quality of life questionnaire v2.1
R: Responders
RP: Role preventive
RR: Role restriction
SAMOHA: Sodium vAlproate in the treatment of Medication Overuse HeadAche
VPA: Sodium valproate.
